# Unsuspected multiples myeloma presenting as bilateral pleural effusion – a cytological diagnosis

**DOI:** 10.1186/1742-6413-4-17

**Published:** 2007-09-07

**Authors:** Kajal Kiran Dhingra, Niti Singhal, Sonu Nigam, Shayama Jain

**Affiliations:** 1 Department of Pathology, Maulana Azad Medical College and Lok Nayak Hospital, Bahadur Shah Zafar Marg, New Delhi 110002, India

## Abstract

**Background:**

Multiple Myeloma presenting as a pleural effusion is extremely rare. It is usually a late complication and is associated with a poor prognosis.

**Case Presentation:**

A 40-year-old male presented with dyspnea and fever of six months duration. Clinical diagnosis of pulmonary tuberculosis was considered. X-ray chest showed bilateral pleural effusion. Pleural cytology revealed numerous plasma cells, some of which were binucleated and atypical. Cytological differential diagnosis included: Myelomatous effusion and Non-Hodgkin's Lymphoma deposit (Immunoblastic type). Bone marrow biopsy, serum protein electrophoresis and bone scan confirmed the diagnosis of multiple myeloma (Plasmablastic type).

**Conclusion:**

Myelomatous pleural effusion as an initial presentation although extremely rare, should always be considered in presence of atypical plasma cells irrespective of age.

## Background

Malignant pleural effusion in Multiple Myeloma (MM) is rare and seen in less than 1% of cases [1]. Identification of the atypical plasma cells in body fluids is important and may be missed when these are scant and mature appearing. Recognition of the atypical plasma cells in fluids is critical for therapeutic and prognostic considerations as this finding portends a poor prognosis [2]. Only eight cases have been reported so far, in which pleural effusion was the initial presentation [2-7]. We report atypical presentation of MM as bilateral pleural effusion in a younger age.

## Case Presentation

**Figure 1:**
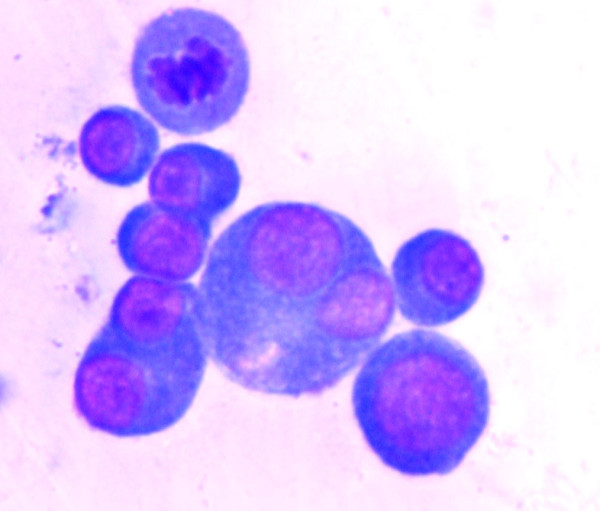
Pleural fluid cytology- Many atypical plasma cells with prominent Nucleoli and binucleate forms are seen. (Giemsa stain × 400).

**Figure 2:**
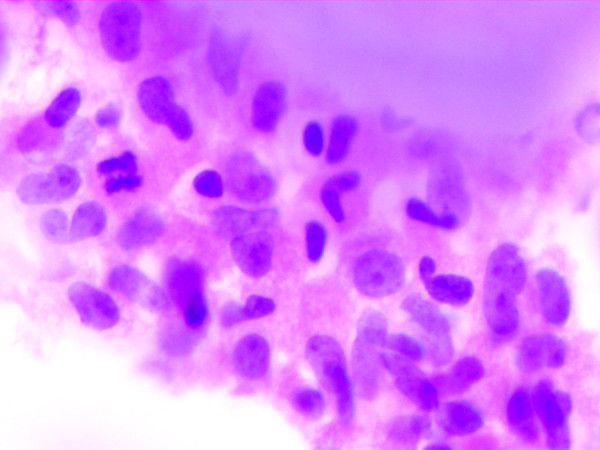
Bone marrow biopsy- sheets of plasma blasts with suppressed Hematopoiesis is seen. (Haematoxylin and Eosin stain × 400).

A 40-year-old male presented with fever and dyspnea of six months duration. The physical examination and chest X-ray was suggestive of bilateral pleural effusion. A diagnostic pleural aspiration was performed from both the sides and cytospin preparation was made. Giemsa stained smears showed cellular smears comprising of many mature and immature plasma cells in a proteinaeous background. These cells had abundant dense blue cytoplasm, a large eccentric nucleus. Frequent binucleated and multinucleate forms, mitotic figures and scattered plasmablasts with prominent nucleoli were also seen (Fig. 1). Thus a diagnosis of Plasma cell dyscrasia versus Non-Hodgkin's Lymphoma (NHL-Immunoblastic type) was suggested. A skeletal survey, serum immunoelectropheresis, bone marrow aspiration and biopsy were advised to confirm the diagnosis. Radiological investigations revealed multiple osteolytic punched out lesions in the axial skeleton. His hemoglobin was 55 g/l; ESR was 85 mm in the first hour by Wintrobe method, and the peripheral smear showed marked rouleax formation. Serum protein electrophoresis showed a sharp M-spike in the IgG region. Bone marrow aspirate smears showed plasmacytoid cells with many binucleate forms. Bone marrow biopsy showed a suppressed hematopoiesis, thinned out bony trabeculae, and near total replacement of the marrow by plasma cells, 50% of which were plasmablastic type (Fig. 2). Based on the above features, a final diagnosis of Multiple Myeloma- Plasmablastic type (MMPT) was made.

## Discussion

Malignant myeloma is a clonal proliferation of plasma cells with multiple osteolytic lesions. It usually occurs in elderly patients (mean age 71 years) and presents with bone pains along with pathological fractures [8]. Malignant pleural effusion is usually a rare and late complication in the course of the disease [9]. Hence other etiologies of reactive pleural effusions like congestive heart failure, pneumonia, tuberculosis, collagen vascular disease, carcinomatosis, AIDS, other viral illness and pulmonary thromboembolism should be excluded before a diagnosis of malignant myelomatous effusion is made [9]. Cytologically these cases can have a predominantly lymphocytic infiltrate with scattered plasma cells showing atypical nuclear features. Differential diagnoses on cytology include other non-myelomatous effusions that present with pleural effusion e.g. NHL, acute and chronic lymphoid leukemias, especially those with concomitant mediastinal involvement [6]. The cytomorphology of the plasma cells along with the clinical profile are helpful in differentiating reactive from malignant plasma cell infiltrates. High cellularity with a predominant plasma cell population in a hemorrhagic or necrotic background favors a malignant effusion. Morphological features of malignant plasma cells are nuclear pleomorphism, prominent nucleoli, frequent mitosis and asynchronous maturation of the nucleus in relation to the cytoplasm. Pleural fluid electrophoresis, flow cytometry and immunocytochemistry aid in confirming the monoclonality of the plasma cells [9].

A malignant effusion in myeloma patients places the patient in advanced Salmon Durie stage. These patients are usually resistant to treatment and often relapse in spite of aggressive chemo-radiotherapy necessitating pleurodesis [2]. This finding suggests a dismal prognosis and death usually ensues within a few months. Therefore, recognition of the atypical plasma cells in the fluid is critical for therapeutic and prognostic considerations [9].

The present case is rare because the diagnosis was unsuspected in younger patient presenting with bilateral pleural effusion. The presence of atypical plasma cells in the body fluids should be carefully interpreted irrespective of the age and, the patient should be thoroughly assessed for MM. Because benign pleural effusions are common in MM patients, the diagnosis of serous cavity involvement by MM is critically important due to poor prognostic implications.

## Conclusion

Myelomatous pleural effusion as an initial presentation although extremely rare, should always be considered in presence of atypical plasma cells irrespective of age. The diagnosis of serous cavity involvement by MM is critical due to poor prognostic implications and fluid cytology forms the mainstay of diagnosis.

## Competing interests

The author(s) declare that they have no competing interests.

## Authors' contributions

Dr Kajal and Dr Niti have assisted in the diagnosis of the case; the cytological work up was done by Dr Shayama Jain. The histopathological diagnosis was made by Dr Sonu Nigam. All authors have contributed in drafting and designing the manuscript and have read and approved of the final manuscript.
